# Response mechanisms of sugarcane seedlings to the allelopathic effects of root aqueous extracts from sugarcane ratoons of different ages

**DOI:** 10.3389/fpls.2022.1020533

**Published:** 2022-09-29

**Authors:** Xiaoming Wang, Shilong Wang, Jinghuan Zhu, Linzhi Zuo, Zuli Yang, Lei Li

**Affiliations:** ^1^ Ministry of Education Key Laboratory for Ecology of Tropical Islands, Hainan Normal University, Haikou, China; ^2^ Key Laboratory for the Green and Efficient Production Technology of Sugarcane, Guangxi Science & Technology Normal University, Laibin, China; ^3^ Laibin Comprehensive Experiment Station of National Sugar Industry Technical System, Laibin Academy of Agricultural Sciences, Laibin, China

**Keywords:** sugarcane seedling, ratoon root system, allelopathy, osmoregulatory substance, enzyme activity

## Abstract

Sugarcane ratoon performance declines with increasing age due to the intergenerational accumulation and release of allelochemicals by old sugarcane roots. We aimed to clarify the effects of these allelochemicals on sugarcane seedling growth under continuous sugarcane ratoon cropping. We investigated the allelopathic effects of treatment with root aqueous extracts from sugarcane ratoons of different ages on the osmoregulatory substance content, antioxidant enzyme activity, membrane lipid peroxidation product content, photosynthetic physiological characteristics, and root exudate enzymatic characteristics of sugarcane seedlings. The root aqueous extracts exerted allelopathic effects on sugarcane seedlings. The relative electrolyte leakage, proline content, soluble protein content, soluble sugar content, malondialdehyde content, and catalase activity of the leaves were more sensitive than those of the roots. Conversely, the superoxide dismutase and peroxidase activities of the roots (positive response) were more sensitive than those of the leaves (negative response). The total chlorophyll content and net photosynthetic rate of the leaves exhibited relatively sensitive negative responses. Urease activity negatively responded but sucrase activity positively responded. We concluded that the root aqueous extracts of sugarcane ratoons may exert their allelopathic effects by affecting the level of osmoregulatory substances and causing peroxidative damage to the cell membranes of sugarcane seedlings and altering the activities of various enzymes in the rhizosphere.

## 1 Introduction

Sugarcane (*Saccharum officinarum*) is a tall and solid, perennial herbaceous plant and C4 crop. It thrives in ample heat and light and is widely distributed in tropical and subtropical regions. China has the third largest sugarcane cultivation area in the world, following Brazil and India. Sugarcane ratooning refers to the practice of harvesting the aboveground portion but leaving the original roots in the soil as the parent plant for a new generation of sugarcane, which grows from the lateral buds of the original roots in the following year. The performance of sugarcane ratoons tends to decline gradually with increasing ratoon age, one of the causes for which may be the intergenerational accumulation and release of allelochemicals in the original root system.

Allelochemicals can alter the physiological properties of crop organs and the physiochemical properties of the rhizosphere (Wang et al., 2013) and can affect the photosynthesis of recipient plants by destroying their cell ultrastructure, which mainly manifests as effects on the plants’ cell membrane system, organelles (e.g., chloroplasts), extracellular secretions, intercellular CO_2_ concentration, antioxidant enzyme activity, and organ function (e.g., transpiration rate) (Liu et al., 2021). For example, growing barley as a companion crop enhanced the activities of protease, urease (UE), and other sucrases (SC) in tomato rhizosphere soil (Yang et al., 2017); companion cropping of tomato with tillered onion increased the activities of polyphenol oxidase (PPO), dehydrogenase, and UE in tomato rhizosphere soil (Wu et al., 2015); allelopathic cultivation of Gynura bicolour promoted the activities of SC, UE, various phosphatases, and PPO in the rhizosphere soil of cowpea under continuous cropping (Chen et al., 2018); allelopathic cultivation of watermelons with garlic as a companion crop promoted peroxidase (POD), PPO, and UE activities and inhibited SC activity (Wu et al., 2021).

Allelopathy and allelopathic substance analysis of sugarcane rhizosphere soil extract on other plants have been tested, while the information about the allelopathy and autotoxicity of sugarcane perennial root extract are limited, and the mechanism of its action on sugarcane seedlings remains a mystery (Hijano et al., 2021). In the present study, sugarcane seedlings propagated from seed canes were used as recipient plants and cultivated hydroponically with the root aqueous extracts of sugar-cane ratoons of different ages. We aimed to simulate the auto toxic effects of allelochemicals released by root system decomposition of sugarcane ratoons on the growth of sugarcane seedlings under natural conditions. We examined the relative electrolyte leakage (REL); proline (Pro), soluble protein (SP), soluble sugar (SS), and malondialdehyde (MDA) contents; and superoxide dismutase (SOD), POD, and catalase (CAT) activities in the leaves and roots of sugarcane seedlings treated with the root aqueous extracts. The responses of total chlorophyll, net photosynthetic rate (*P*_n_), intercellular CO_2_ concentration (*C*_i_), and transpiration rate (*T*_r_) in the leaves of sugarcane seedlings and the response patterns of POD, PPO, acid phosphatase (ACP), UE, and SC activities in the culture medium were analysed. We aimed to explore the effects of the root aqueous extracts from sugarcane ratoons on the content of osmoregulatory substances, antioxidant enzyme activities, lipid peroxidation products, photosynthetic physiological characteristics, and root exudate enzymatic characteristics of sugarcane seedlings. Our findings will provide a theoretical basis for understanding the mechanisms of allelopathic autotoxicity during the decline in sugarcane ratoon performance.

## 2 Materials and methods

### 2.1 Plant materials

For the sugarcane roots, first-, second-, and third-year sugarcane ratooning plots cultivating the Guiliu 05/136 variety were selected from the Liangtang “double high sugarcane” land consolidation zone (109°E, 23°N) in Xingbin District, Laibin City, Guangxi Province. A five-point sampling method was employed to select and excavate sugarcane roots. The aboveground portion was removed and the belowground portion was retained; care was taken to maintain the integrity of the sugarcane root system. After gently shaking off the soil and impurities, the roots were placed in a sample bag and returned to the laboratory for further processing.

For sugarcane seed canes, non-commercially ripened, healthy, whole-stem sugarcane seed canes above 1.2 m were obtained from newly cultivated sugarcane plots growing the Guiliu 05/136 variety in the abovementioned zone. The mass of sugarcane seed canes was kept constant where possible. Each seed cane was divided into segments containing one bud each and cuts were made 2.5 cm above and below each node in preparation for later use.

### 2.2 Experimental methods

#### 2.2.1 Preparation of sugarcane root aqueous extracts

The roots were carefully brushed with a sterile brush to remove adhering soil. After air drying or oven drying below 45°C, the samples were cut into small pieces of less than 2 cm, mechanically pulverised, and passed through a 40-mesh sieve. Subsequently, 60 g of sieved sample was weighed into a conical flask, 1000 mL of ultrapure water was added, and the flask was sealed tight and placed in an incubator shaker for 5 d (temperature: 25°C, shaking speed: 100 r/min). Suction filtration was performed using a vacuum pump to obtain stock solutions (concentration: 60 g·L^−1^) for sugarcane root aqueous extracts of different ratoon ages. The root aqueous extracts for first-, second-, and third-year sugarcane ratoons were labelled as J1, J2, and J3, respectively. Further dilution was performed using ultrapure water to obtain extracts with concentrations of 20 and 40 g·L^−1^, which were then stored at 4°C for later use. Using the J1 extracts as an example, the three different levels in the concentration gradient (20, 40, and 60 g·L^−1^) were labelled as J1-1, J1-2, and J1-3, respectively. The control (CK) was treated with ultrapure water.

#### 2.2.2 Experimental treatments

Sugarcane seed canes were germinated and cultivated in a programme-controlled artificial climate chamber (temperature 32°C, humidity 70%, light 10 h·d^−1^, dark 14 h·d^−1^, cultivation duration 10 d). Germination was carried out in germination trays (length × width × height: 50 × 30 × 4 cm). The seed canes were first immersed in 1% formaldehyde solution for 20 min. The germination trays were lined with two layers of sterile qualitative filter papers; 20 buds were placed in each tray and approximately 1000 mL of ultrapure water was added. Finally, the trays were covered with two layers of polyethylene wrap to preserve moisture. Sugarcane seedlings exhibiting consistent vigour were selected for transplantation to germination trays containing equal volumes of full-concentration Hoagland nutrient solution. After cultivating for 10 d, the sugarcane seedlings were transplanted into germination trays containing 1000 mL of equal amounts of sugarcane root extracts. After treatment with different concentrations of aqueous extracts for 10 d, samples were collected to determine various indicators.

### 2.3 Measurement indicators and methods

#### 2.3.1 Physiological indicators of sugarcane leaves and roots

The REL was measured by referencing the method described in *Experimental Guidance for Plant Physiology* (Zhang et al., 2009). The Pro content was determined using the sulfosalicylic acid colorimetric method (Wu et al., 2021). The SP content was determined using the Coomassie brilliant blue method (Wu et al., 2021). The SS content was determined using the phenol-sulphuric acid method (Wu et al., 2021). The MDA content was determined using the thiobarbituric acid heated colorimetric method (Li and Zhang, 2016). The SOD activity was measured using the mitroblue tetrazolium (NBT) method (reagent kit method), and the amount of enzyme required to inhibit NBT reduction to 50% of the control was defined as one unit of enzyme activity (U). The activity of POD was determined using a micro glass cuvette colorimetric (purpurogallin) assay kit, for which every 1 mg purpurogallin produced per gram of sample per day was defined as 1 unit of enzyme activity (U). Further, CAT activity was determined using the same kit, for which every 1 μmol of H_2_O_2_ degraded per gram of sample per day was defined as 1 U.

#### 2.3.2 Physiological indicators of photosynthesis

Total chlorophyll content was measured with reference to Zhang (1992). *P*_n_, *C*_i_, and *T*_r_ were measured using a portable photosynthesis system (LI-COR US).

#### 2.3.3 Enzyme activity indicators in culture medium

Enzyme activity was determined using the micro method. The activity of POD was determined using the guaiacol colorimetric method, for which every 0.01 change in absorbance per minute was defined as 1 U. Importantly, PPO activity was determined using a pyrogallol colorimetric assay kit, for which every 1 mg of purpurogallin produced per gram of sample per day was defined as 1 U. The ACP activity was determined using a disodium phenyl phosphate assay kit, for which every 1 nmol of phenol produced per gram of sample per day was defined as 1 U. Further, UE activity was determined using an indophenol blue colorimetric assay kit, for which every 1 μg of NH_3_-N produced per gram of sample per day was defined as 1 U. Moreover, SC activity was determined using a 3,5-dinitrosalicylic acid colorimetric assay kit, for which every 1 mg of reducing sugar produced per gram of sample per day was defined as 1 U.

### 2.4 Statistical analysis

Significant differences were analysed using Minitab 16. Graphing was performed using Origin 2018. A two-way analysis of variance was performed using SPSS 22.0. Data were processed using Excel 2016. All experimental data are expressed as mean ± standard deviation.

## 3 Results and Analysis

### 3.1 Responses of osmoregulatory substance content

As shown in **Tables 1** and **2**, the leaf and root REL of sugarcane seedlings responded differently to different concentrations of root aqueous extracts from sugarcane ratoons of different ages. The root aqueous extracts of sugarcane ratoons for all three years induced an upward trend in the leaf and root REL of sugarcane seedlings with increasing treatment concentration. Under treatment with 40 and 60 g·L^−1^ root aqueous extracts from sugarcane ratoons of all three years, the leaf REL was significantly greater than that in the control (CK) (*P* < 0.05), whereas the root REL was only significantly greater than that in CK (*P* < 0.05) for third-year ratoons. Root REL was only significantly greater than that in CK (*P <* 0.05) across all three years under 60 g·L^−1^ root aqueous extract treatment. J2-3 produced the highest REL for the leaves and roots (153.8% and 131.3% of that in CK, respectively). In response to treatment with aqueous extracts of increasing ratoon age, REL exhibited an overall trend of initially increasing before decreasing. Based on these findings, The leaf REL was more sensitive to the root aqueous extracts of sugarcane ratoons, with positive responses in all cases, and J2-3 significantly increased REL compared with CK (*P <* 0.05).

**Table 1:** Responses of osmoregulatory substance content in the leaves of sugarcane seedlings to different root aqueous extracts.

Treatment andconcentration (g·L^−1^)		REL (%)	Pro content (μg·g^−1^)	SP content(mg·g^−1^)	SS content(mg·g^−1^)
CK	0	26.16 ± 2.40c	86.81 ± 5.43a	71.38 ± 6.11a	11.23 ± 1.29c
J1-1	20	28.67 ± 1.39bc	74.57 ± 5.35b	60.96 ± 2.66b	13.27 ± 1.10bc
J1-2	40	32.51 ± 3.44b	81.04 ± 6.78ab	53.34 ± 6.14bc	14.29 ± 1.34ab
J1-3	60	38.42 ± 1.76a	92.25 ± 7.37a	48.76 ± 3.52c	16.16 ± 1.39a
CK	0	26.16 ± 2.40c	86.81 ± 5.43b	71.38 ± 6.11a	11.23 ± 1.29b
J2-1	20	30.81 ± 2.96bc	64.66 ± 8.70c	62.44 ± 2.61ab	12.85 ± 0.88b
J2-2	40	33.86 ± 3.13b	84.21 ± 5.05b	57.07 ± 4.06b	15.21 ± 0.55a
J2-3	60	40.23 ± 3.18a	102.08 ± 8.99a	54.22 ± 5.48b	16.48 ± 0.87a
CK	0	26.16 ± 2.40c	86.81 ± 5.43a	71.38 ± 6.11a	11.23 ± 1.29b
J3-1	20	27.15 ± 2.00bc	75.53 ± 4.91b	62.56 ± 7.06ab	12.26 ± 0.68b
J3-2	40	32.13 ± 3.62ab	83.83 ± 3.55ab	60.42 ± 6.19ab	12.96 ± 0.92b
J3-3	60	37.29 ± 3.53a	93.93 ± 8.71a	54.35 ± 3.49b	15.27 ± 1.09a

Different lowercase letters in each column represent significant differences at the 5% level between different concentrations of root aqueous extract with the same ratoon age. REL, relative electrolyte leakage; Pro, proline; SP, soluble protein; SS, soluble sugar; CK, control.

**Table 2:** Responses of osmoregulatory substances in the roots of sugarcane seedlings to different root aqueous extracts.

Treatment and concentration (g·L^−1^)		REL (%)	Pro content (μg·g^−1^)	SP content(mg·g^−1^)	SS content(mg·g^−1^)
CK	0	22.97 ± 1.57b	88.69 ± 2.02b	80.88 ± 2.42a	17.52 ± 1.22c
J1-1	20	23.03 ± 2.01b	90.97 ± 2.44b	80.16 ± 2.61a	18.19 ± 1.32bc
J1-2	40	23.68 ± 0.99b	98.22 ± 3.03a	77.51 ± 2.05a	19.96 ± 1.46ab
J1-3	60	26.64 ± 1.16a	92.79 ± 2.62b	69.35 ± 2.78b	20.89 ± 1.00a
CK	0	22.97 ± 1.57b	88.69 ± 2.02c	80.88 ± 2.42a	17.52 ± 1.22c
J2-1	20	25.19 ± 1.92b	102.73 ± 2.99b	78.02 ± 2.37a	19.19 ± 1.20bc
J2-2	40	25.51 ± 1.06b	115.16 ± 2.40a	70.40 ± 1.99b	21.21 ± 1.51ab
J2-3	60	30.16 ± 1.18a	87.94 ± 2.57c	66.71 ± 1.50b	22.75 ± 0.95a
CK	0	22.97 ± 1.57b	88.69 ± 2.02c	80.88 ± 2.42a	17.52 ± 1.22bc
J3-1	20	21.93 ± 1.50b	93.01 ± 2.43b	85.08 ± 3.10a	15.82 ± 1.43c
J3-2	40	27.63 ± 1.64a	97.10 ± 2.56a	72.29 ± 2.00b	18.93 ± 1.17ab
J3-3	60	27.01 ± 1.65a	92.82 ± 1.45b	75.28 ± 2.40b	20.59 ± 1.40a

Different lowercase letters in each column represent significant differences at the 5% level between different concentrations of root aqueous extract with the same ratoon age. REL, relative electrolyte leakage; Pro, proline; SP, soluble protein; SS, soluble sugar; CK, control

The Pro content in the leaves and roots exhibited inconsistent responses to different concentrations of root aqueous extracts from sugarcane ratoons of different ages. In the leaves, Pro content increased with increasing extract concentration and was significantly lower in the 20 g·L^−1^ treatment than in CK (*P <* 0.05) for all ratoon ages. Pro content in the 60 g·L^−1^ treatment was greater than that in CK (but only J2-3 showed a significant difference (*P <* 0.05)). In the roots, Pro content showed a trend of initially increasing before decreasing with increasing extract concentration and was greater in the 40 g·L^−1^ treatment compared with that in CK for all ratoon ages, but this was only significant for all concentrations when treated with the aqueous extract of third-year ratoons (*P <* 0.05). J2-1 produced the lowest Pro content in the leaves (74.5% of that in CK) and J2-3 produced the highest Pro content in the roots (129.8% of that in CK). The response of Pro content to treatment with root aqueous extracts of different ratoon ages was second-year extract > first- and third-year extract. Therefore, the Pro content in the leaves of sugarcane seedlings was more sensitive than that in the roots to the root aqueous extracts of sugarcane ratoons, with leaf Pro content generally showing negative responses and root Pro content showing positive responses.

The SP content in the leaves and roots decreased with increasing extract concentration. However, leaf SP content increased with increasing ratoon age, whereas root SP content decreased initially before increasing with increasing ratoon age. When treated with 60 g·L^−1^ root aqueous extracts, the SP content of both leaves and roots was lower than that in CK (*P <* 0.05). Leaf SP content was more sensitive to all concentrations of first-year extract, all of which significantly decreased SP content compared with CK (*P <* 0.05). However, root SP content was more sensitive to treatment with 40 and 60 g·L^−1^ second- and third-year extracts, all of which significantly decreased SP content compared with CK (*P <* 0.05). J1-1 produced the lowest SP content in the leaves (68.3% of that in CK) and J2-3 produced the lowest SP content in the roots (82.5% of that in CK). Based on the above, the SP content in the leaves was more sensitive than that in the roots to the root aqueous extracts of sugarcane ratoons, with negative responses overall, and J1-3 significantly decreased SP content compared with CK (*P <* 0.05).

The SS content in the leaves and roots increased with increasing extract concentration while also showing a trend of initially increasing before decreasing with increasing ratoon age. When plants were treated with 40 and 60 g·L^−1^ root aqueous extract, the SS content in both leaves and roots was significantly greater than that in CK (*P <* 0.05) (except for J3-2). Furthermore, the SS content of both the leaves and roots was sensitive to all concentrations of first- and second-year extracts, all of which increased SS content compared with CK. J2-3 produced the highest SS content in the leaves and roots (146.7% and 129.8% of that in CK, respectively). Therefore, the SS content in the leaves was more sensitive than that in the roots to the root aqueous extracts of sugarcane ratoons, with positive responses overall, and J2-3 significantly increased SS content compared with CK (*P <* 0.05).

### 3.2 Responses of antioxidant enzyme activity and membrane lipid peroxidation product content

As shown in **Tables 3** and **4**, the MDA content in the leaves and roots generally increased with increasing extract concentration while also showing a general trend of decreasing with increasing ratoon age. In the leaves, MDA content was significantly greater than that in CK (*P <* 0.05) for all concentrations only when plants were treated with first-year root aqueous extracts and only 60 g·L^−1^ third-year extracts elicited a significant response. In the roots, MDA content was significantly greater than that in CK (*P <* 0.05) when plants were treated with 40 and 60 g·L^−1^ first-year extracts but only 40 g·L^−1^ third-year extracts. J1-3 produced the highest MDA content in the leaves and roots (150.6% and 118.6% of that in CK, respectively). Therefore, the MDA content in the leaves was more sensitive than that in roots to the root aqueous extracts of sugarcane ratoons, with positive responses in all cases, and J1-3 significantly increased MDA content compared with CK (*P <* 0.05).

**Table 3:** Responses of antioxidant enzyme activity and membrane lipid peroxidation product content in the leaves of sugarcane seedlings to different root aqueous extracts.

Treatment and concentration (g·L^−1^)		MDA content (nmol·g^−1^)	SOD activity (U·g^−1^)	POD activity (U·g^−1^)	CAT activity (U·g^−1^)
CK	0	12.40 ± 0.96c	280.4 ± 10.0bc	495.1 ± 18.4a	62.7 ± 4.5ab
J1-1	20	15.70 ± 1.58b	290.2 ± 9.5b	473.7 ± 11.3a	68.7 ± 5.1a
J1-2	40	18.42 ± 1.81a	313.2 ± 3.1a	437.0 ± 16.6b	54.0 ± 4.4bc
J1-3	60	18.68 ± 1.17a	267.4 ± 8.8c	403.2 ± 17.0c	48.4 ± 5.7c
CK	0	12.40 ± 0.96b	280.4 ± 10.0b	495.1 ± 18.4a	62.7 ± 4.5a
J2-1	20	12.61 ± 1.31b	283.9 ± 7.8b	462.8 ± 19.1a	70.9 ± 5.4a
J2-2	40	17.94 ± 1.62a	298.8 ± 10.0a	430.7 ± 13.3b	51.3 ± 4.2b
J2-3	60	18.41 ± 1.89a	269.8 ± 4.0b	384.1 ± 10.4c	43.2 ± 3.9b
CK	0	12.40 ± 0.96b	280.4 ± 10.0a	495.1 ± 18.4a	62.7 ± 4.5a
J3-1	20	10.97 ± 1.12b	296.2 ± 10.6a	474.5 ± 13.4ab	62.2 ± 5.6a
J3-2	40	11.95 ± 1.14b	297.6 ± 9.3a	462.6 ± 12.5b	55.7 ± 5.0ab
J3-3	60	15.34 ± 1.08a	282.1 ± 6.5a	419.5 ± 15.4c	50.8 ± 4.3b

Different lowercase letters in each column represent significant differences at the 5% level between different concentrations of root aqueous extract with the same ratoon age. MDA, malondialdehyde; SOD, superoxide dismutase; POD, peroxidase; CAT, catalase; CK, control.

**Table 4:** Responses of antioxidant enzyme activity and membrane lipid peroxidation product content in the roots of sugarcane seedlings to different root aqueous extracts.

Treatment and concentration (g·L^−1^)		MDA content (nmol·g^−1^)	SOD activity (U·g^−1^)	POD activity (U·g^−1^)	CAT activity (U·g^−1^)
CK	0	13.09 ± 1.19b	254.9 ± 10.8d	40.5 ± 5.0c	19.2 ± 1.6b
J1-1	20	13.58 ± 1.12ab	279.0 ± 10.1c	74.4 ± 6.1b	19.2 ± 1.9b
J1-2	40	15.43 ± 0.93a	330.5 ± 9.6b	87.5 ± 6.5b	21.2 ± 2.0ab
J1-3	60	15.53 ± 1.57a	383.8 ± 13.3a	119.3 ± 9.6a	22.6 ± 1.6a
CK	0	13.09 ± 1.19b	254.9 ± 10.8d	40.5 ± 5.0d	19.2 ± 1.6b
J2-1	20	14.35 ± 0.98ab	282.5 ± 17.5c	82.9 ± 8.8c	20.7 ± 1.7ab
J2-2	40	14.75 ± 1.25ab	344.1 ± 13.2b	109.4 ± 7.2b	23.5 ± 1.4a
J2-3	60	15.51 ± 1.51a	397.8 ± 15.5a	167.1 ± 9.2a	23.6 ± 1.7a
CK	0	13.09 ± 1.19b	254.9 ± 10.8c	40.5 ± 5.0c	19.2 ± 1.6a
J3-1	20	12.26 ± 1.06b	267.5 ± 16.6bc	48.8 ± 5.3bc	20.4 ± 1.5a
J3-2	40	15.30 ± 0.98a	289.3 ± 14.2b	59.3 ± 5.2b	21.5 ± 1.9a
J3-3	60	12.88 ± 1.06b	334.3 ± 15.0a	103.8 ± 7.5a	21.6 ± 1.8a

Different lowercase letters in each column represent significant differences at the 5% level between different concentrations of root aqueous extract with the same ratoon age. MDA, malondialdehyde; SOD, superoxide dismutase; POD, peroxidase; CAT, catalase; CK, control.

The SOD activity in the leaves and roots exhibited inconsistent responses to treatment with different concentrations of aqueous root extracts from sugarcane ratoons of different ages. In the leaves, SOD activity showed a general trend of initially increasing before decreasing with increasing extract concentration. However, in the roots, SOD activity increased with increasing extract concentration. Furthermore, SOD activity in the leaves showed a trend of initially decreasing before increasing with increasing ratoon age, whereas that in the roots showed a trend of initially increasing before decreasing with increasing ratoon age. Leaf SOD activity was significantly greater than that in CK (*P <* 0.05) only when plants were treated with 40 g·L^−1^ first- and second-year extracts. Root SOD activity was significantly greater than that in CK (*P <* 0.05) when plants were treated with all concentrations of first- and second-year extracts and 40 g·L^−1^ and 60 g·L^−1^ third-year extracts. J1-2 produced the highest SOD activity in the leaves (111.7% of that in CK); J2-3 produced the highest SOD activity in the roots (156.1% of that in CK). These findings indicate that the SOD activity in the roots was more sensitive than that in leaves to treatment with the root aqueous extracts of sugarcane ratoons, with leaves responding positively to low concentrations and negatively to high concentrations, and the roots responding positively overall. In addition, SOD activity was significantly greater under J2-3 compared with that in CK (*P <* 0.05).

The POD activity in the leaves decreased with increasing extract concentration, while that in the roots increased with increasing extract concentration. In addition, leaf POD activity showed a general trend of initially decreasing before increasing with increasing ratoon age, while root POD activity showed a general trend of initially increasing before decreasing with increasing ratoon age. In the leaves, POD activity was significantly lower than that in CK (*P <* 0.05) only when plants were treated with 40 and 60 g·L^−1^ extracts of different ratoon ages. In the roots, POD activity was significantly greater than that in CK (*P <* 0.05) when plants were treated with all concentrations of first- and second-year extracts. J2-3 produced the lowest POD activity in the leaves and the highest POD activity in the roots (44.6% and 412.6% of that in CK, respectively). Therefore, POD activity in the roots was more sensitive than that in leaves to treatment with the root aqueous extracts of sugarcane ratoons, with the leaves showing negative responses and the roots showing positive responses overall. Furthermore, leaf POD activity was significantly decreased, and root POD activity increased under J2-3 compared with those in CK (*P <* 0.05).

The CAT activity in the leaves decreased with increasing extract concentration, while that in the roots increased with increasing extract concentration. In addition, leaf CAT activity showed a general trend of decreasing with increasing ratoon age, while root CAT activity showed a general trend of initially increasing before decreasing with increasing ratoon age. In the leaves, CAT activity was significantly lower than that in CK (*P <* 0.05) when plants were treated with 60 g·L^−1^ extracts of different ratoon ages. In the roots, CAT activity was significantly greater than that in CK (*P <* 0.05) under J1-3, as well as J2-2 and J2-3. J2-3 produced the lowest CAT activity in the leaves and the highest CAT activity in the roots (68.9% and 122.9% of that in CK, respectively). In particular, CAT activity in the leaves and roots was significantly lower and higher under J2-3 compared with that in CK (*P <* 0.05), respectively.

### 3.3 Responses of photosynthetic physiological characteristics

**Figure 1:**
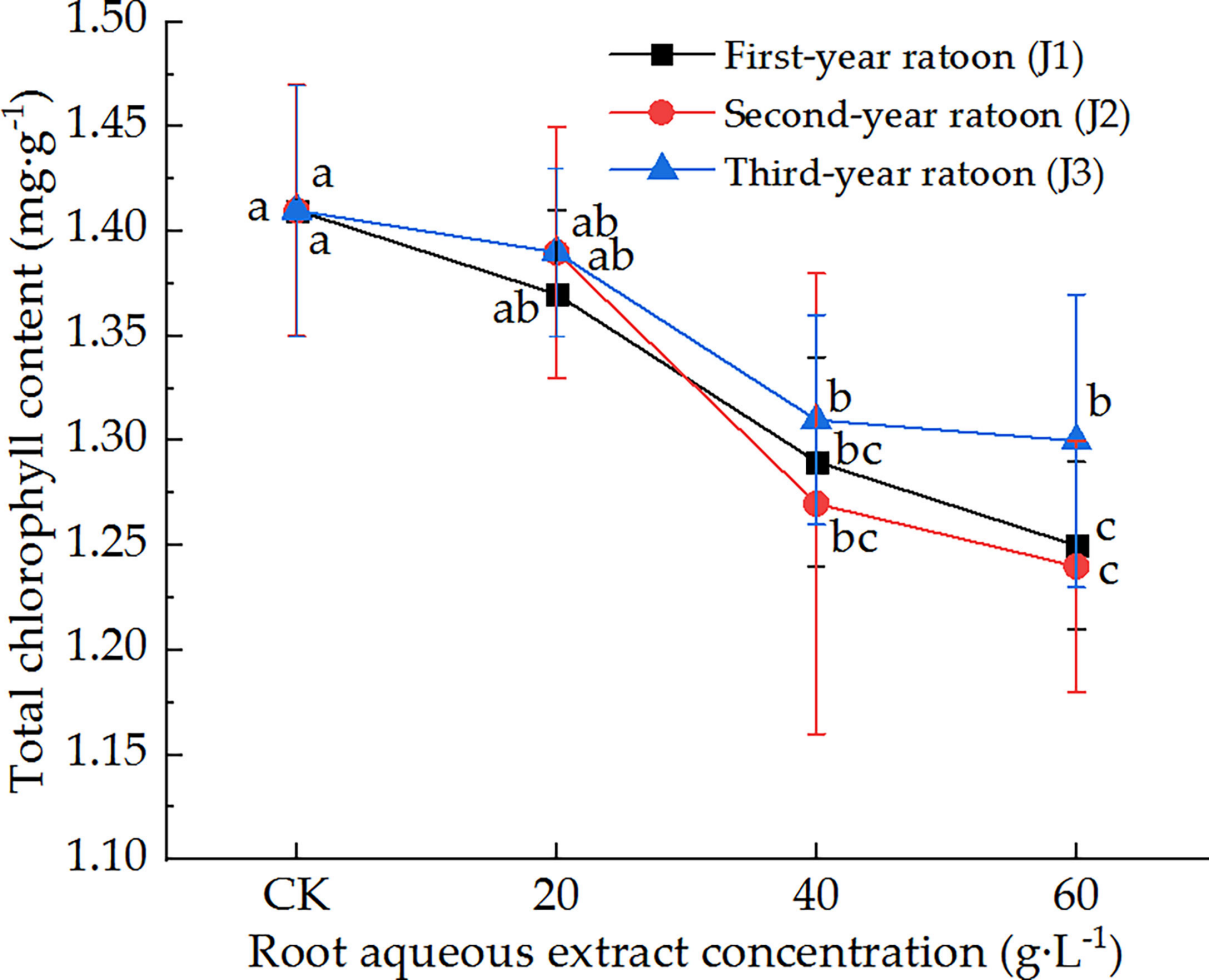
Response of total chlorophyll content in sugarcane seedlings to different root aqueous extracts. Different lowercase letters represent significant differences at the 5% level between different concentrations of root aqueous extract with the same ratoon age.

As shown in **Figure 1**, the total chlorophyll content in the leaves decreased with increasing extract concentration and showed a general upward trend with increasing ratoon age. Total chlorophyll content was significantly lower than that in CK (*P <* 0.05) for different ratoon ages when plants were treated with 40 and 60 g·L^−1^ root aqueous extract. J1-3 and J2-3 significantly decreased total chlorophyll content compared with that in J1-1 and J2-1 (*P <* 0.05). Total chlorophyll content was lower than that in CK for all concentrations when plants were treated with third-year extracts but the differences were not significant. J2-3 led to the lowest total chlorophyll content in the leaves (87.9% of that in CK). Therefore, we can conclude that the total chlorophyll content in the leaves of sugarcane seedlings was sensitive to treatment with the root aqueous extracts of sugarcane ratoons, while its response to different ratoon ages was as follows: second-year extract > first-year extract > third-year extract.

**Figure 2:**
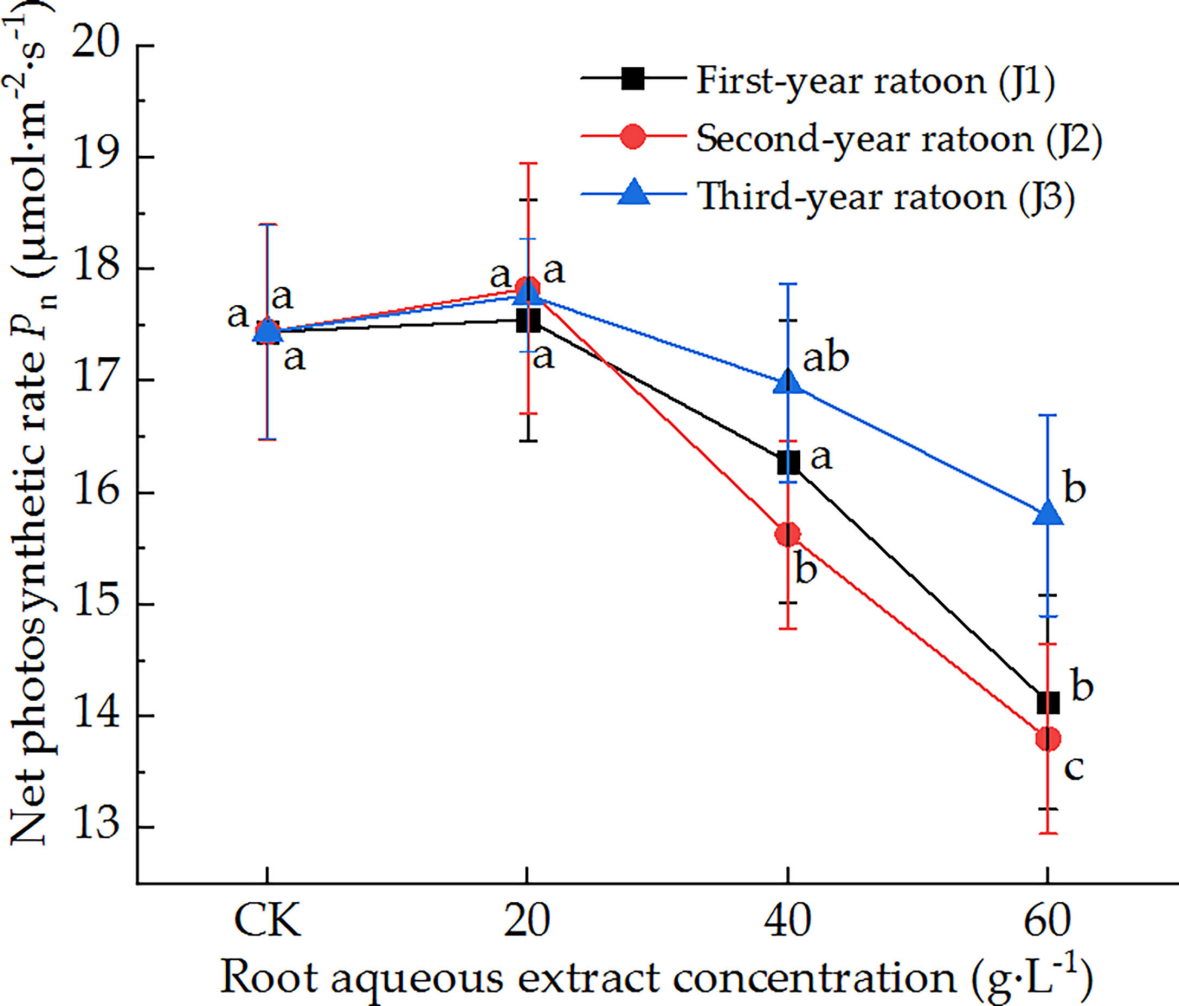
Response of *P*_n_ in sugarcane seedlings to different root aqueous extracts. Different lowercase letters represent significant differences at the 5% level between different concentrations of root aqueous extract with the same ratoon age.

As shown in **Figure 2**, the leaf *P*_n_ of sugarcane seedlings decreased with increasing extract concentration and showed a general trend of initially decreasing before increasing with increasing ratoon age. *P*_n_ was significantly lower than that in CK (*P <* 0.05) under treatment with 60 g·L^−1^ root aqueous extract for all ratoon ages but was greater than that in CK (non-significant difference) when plants were treated with 20 g·L^−1^ root aqueous extract for all ratoon ages. However, only the second-year extract showed a significant difference with the different concentrations. J2-3 led to the lowest *P*_n_ (79.1% of that in CK). Based on these findings, we conclude that the leaf *P*_n_ showed a relatively sensitive negative response to treatment with the root aqueous extracts of sugarcane ratoons, while its response to the root aqueous extracts of different ratoon ages was as follows: second-year extract > first-year extract > third-year extract.

**Figure 3:**
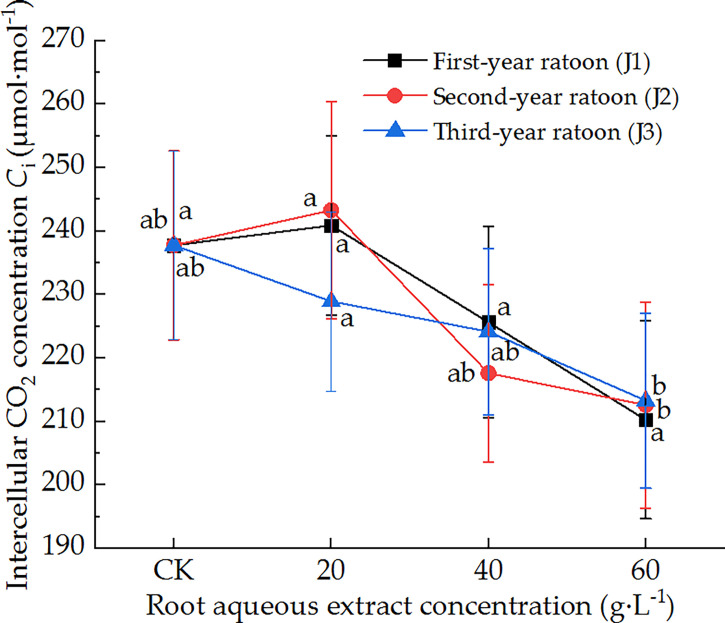
Response of *C*_i_ in sugarcane seedlings to different root aqueous extracts. Different lowercase letters represent significant differences at the 5% level between different concentrations of root aqueous extract with the same ratoon age.

As shown in **Figure 3**, the leaf *C*_i_ of sugarcane seedlings decreased with increasing extract concentration and did not vary significantly with increasing ratoon age. *C*_i_ did not differ significantly from that in CK for all concentrations of root aqueous extracts with different ratoon ages. J1-1 and J2-1 significantly increased *C*_i_ compared with J1-3 and J2-3 (*P <* 0.05). J1-3 produced the lowest *C*_i_ (88.4% of that in CK). Therefore, these findings indicate that leaf *C*_i_ was not sensitive to different concentrations of root aqueous extracts with different ratoon ages.

**Figure 4:**
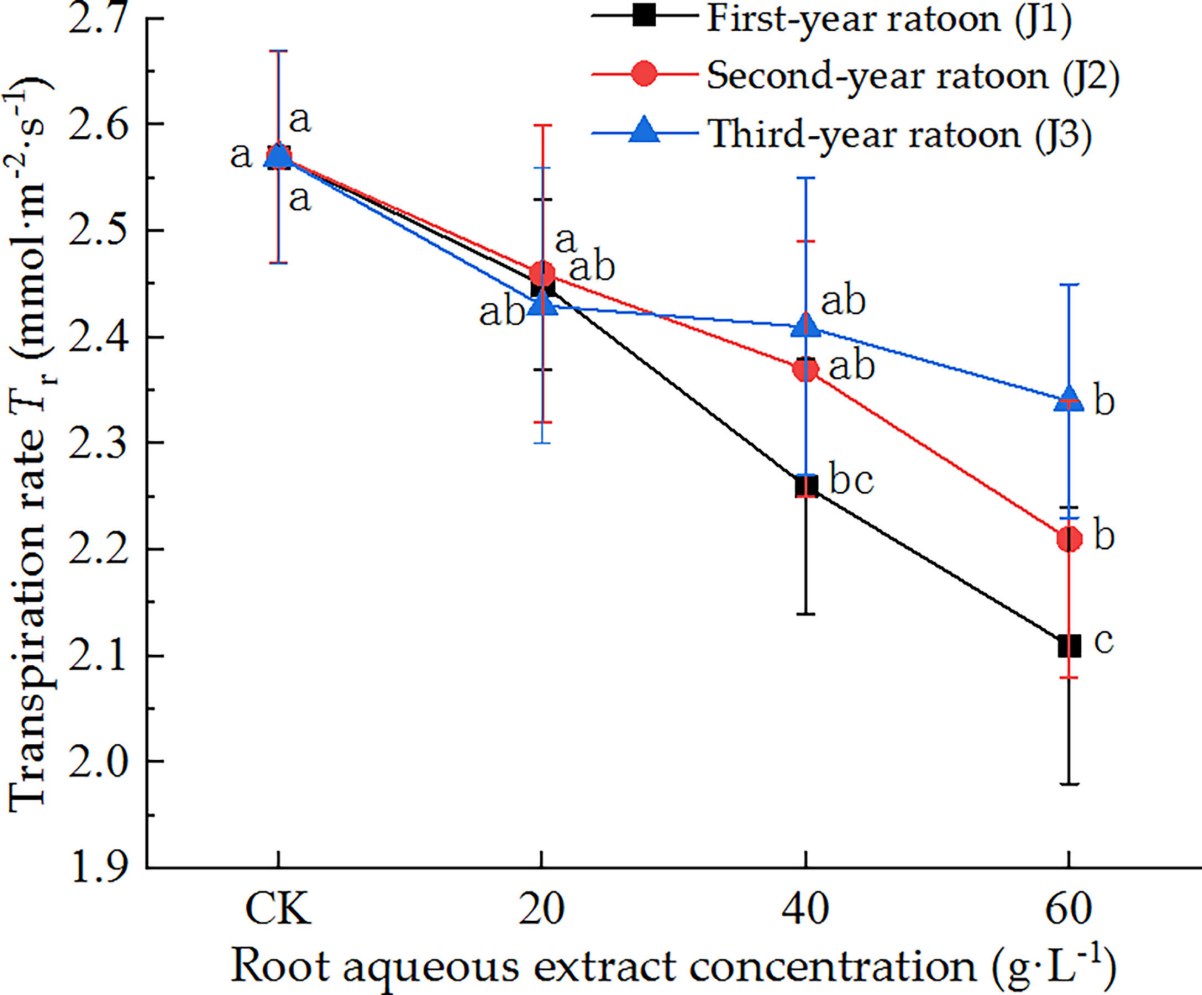
Response of *T*_r_ in sugarcane seedlings to different root aqueous extracts. Different lowercase letters represent significant differences at the 5% level between different concentrations of root aqueous extract with the same ratoon age.

As shown in **Figure 4**, the leaf *T*_r_ decreased with increasing extract concentration and increased with increasing ratoon age. *T*_r_ was significantly lower than that in CK (*P <* 0.05) under treatment with 60 g·L^−1^ root aqueous extract for all ratoon ages, and non-significantly lower under J2-1, J2-2, J3-1, and J3-2. J1-3 produced the lowest *T*_r_ (82.1% of that in CK). The leaf *T*_r_ showed negative responses to treatment with the root aqueous extracts of sugarcane ratoons and was more sensitive to the extracts of first-year ratoons. Finally, J1-2 significantly decreased *T*_r_ compared with CK (*P <* 0.05).

### 3.4 Responses of enzymatic characteristics

**Figure 5:**
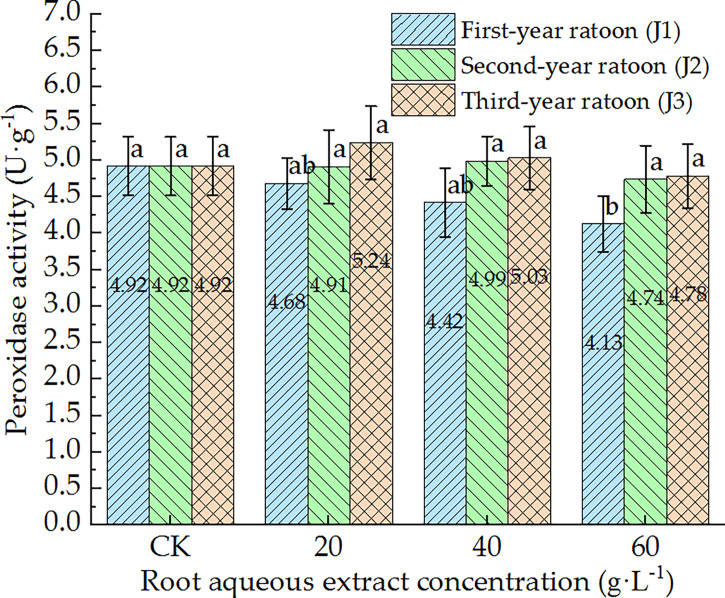
Response of POD activity in the culture medium of sugarcane seedlings to different root aqueous extracts. Different lowercase letters represent significant differences at the 5% level between different concentrations of root aqueous extract with the same ratoon age.

**Figure 6:**
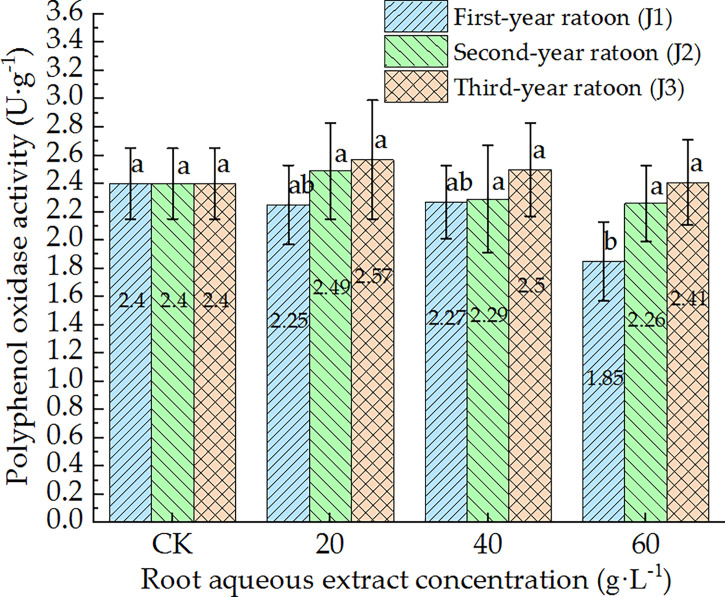
Response of PPO activity in the culture medium of sugarcane seedlings to different root aqueous extracts. Different lowercase letters represent significant differences at the 5% level between different concentrations of root aqueous extract with the same ratoon age.

**Figure 7:**
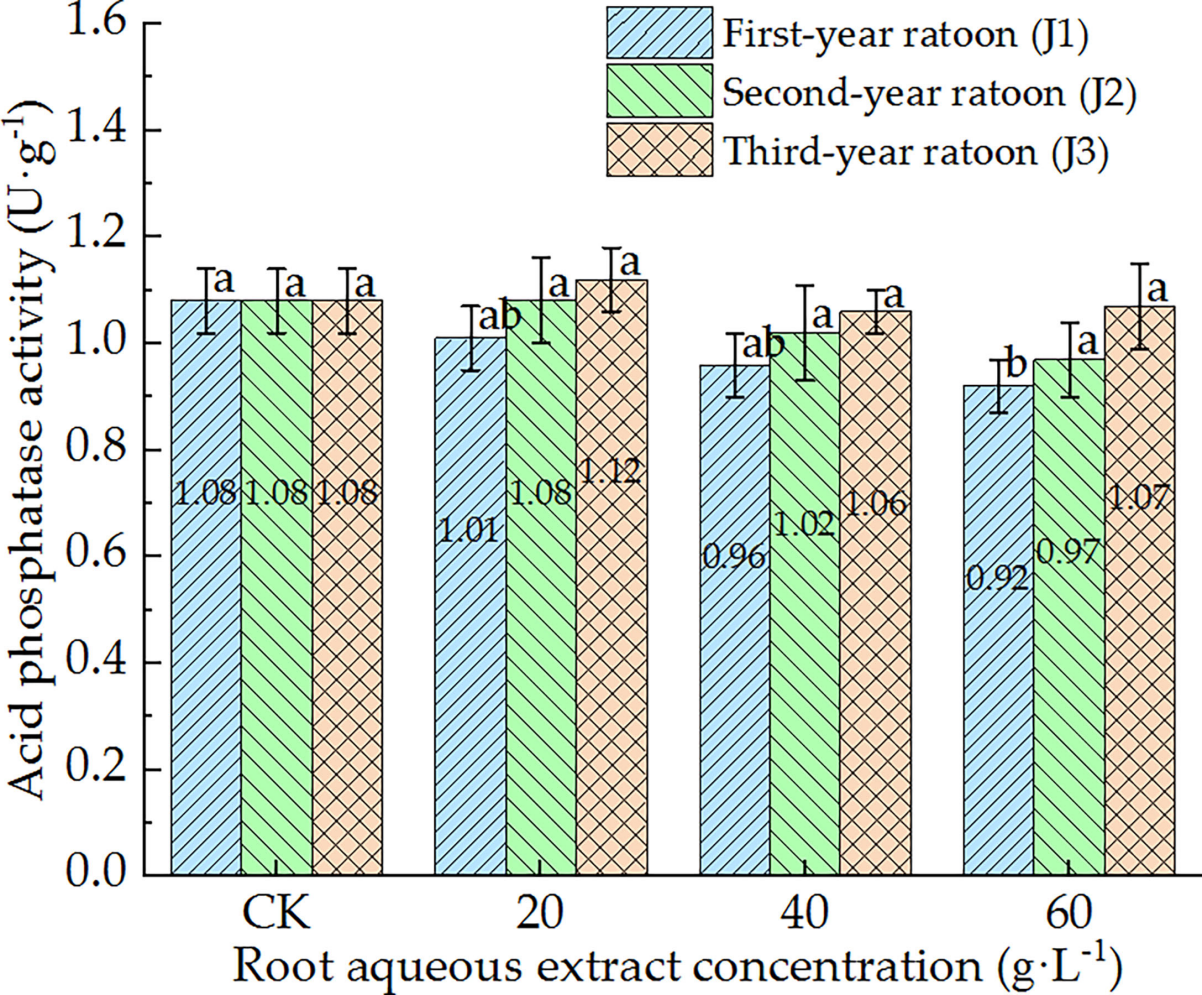
Response of ACP activity in the culture medium of sugarcane seedlings to different root aqueous extracts. Different lowercase letters represent significant differences at the 5% level between different concentrations of root aqueous extract with the same ratoon age.

As shown in **Figures 5**–**7**, POD, PPO, and ACP activities were consistent in the culture medium, all of which decreased with increasing extract concentration and showed a general trend of gradually increasing with increasing ratoon age. POD, PPO, and ACP activities were only significantly lower than those in CK (*P <* 0.05) under J1-3; POD and PPO activities were greater than those in CK (non-significant difference) under J3-1 and J3-2; ACP activity was greater than that in CK (non-significant difference) under J3-1. The differences among the different extract concentrations were not significant for all ratoon ages. J1-3 produced the lowest POD, PPO, and ACP activities (83.9%, 77.1%, and 85.2% of that in CK, respectively). Therefore, the POD, PPO, and ACP activities in the culture medium generally exhibited negative responses to treatment with the root aqueous extracts of sugarcane ratoons. However, the responses were not sensitive and were only significantly lower than those in CK (*P <* 0.05) under J1-3, while also showing greater sensitivity to first-year extracts compared to those of other years.

**Figure 8:**
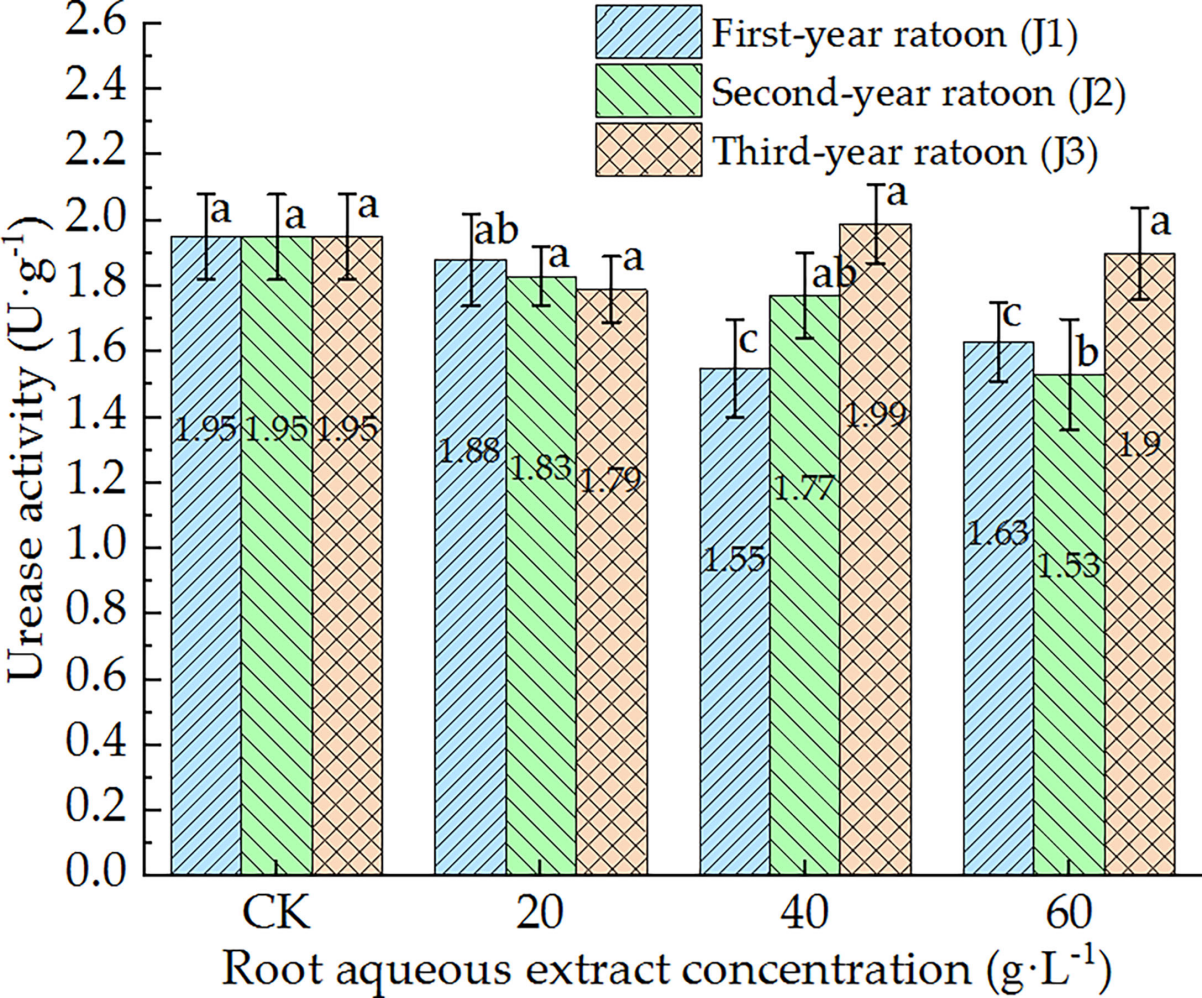
Response of UE activity in the culture medium of sugarcane seedlings to different root aqueous extracts. Different lowercase letters represent significant differences at the 5% level between different concentrations of root aqueous extract with the same ratoon age.

As shown in **Figure 8**, UE activity in the culture medium decreased with increasing extract concentration and showed a general trend of gradually increasing with increasing ratoon age. UE activity was significantly lower than that in CK (*P <* 0.05) only under J1-2, J1-3, and J2-3; was greater than that in CK (non-significant difference) under J3-2; showed no significant differences among the different concentrations when treated with third-year extracts. J2-3 led to the lowest UE activity (78.5% of that in CK). Therefore, these findings indicate that UE activity in the culture medium generally exhibited negative responses to treatment with the root aqueous extract of sugarcane ratoons. In particular, UE activity was significantly lower than that in CK and 20 g·L^−1^ treatment (*P <* 0.05) under J1-2 and J1-3.

**Figure 9:**
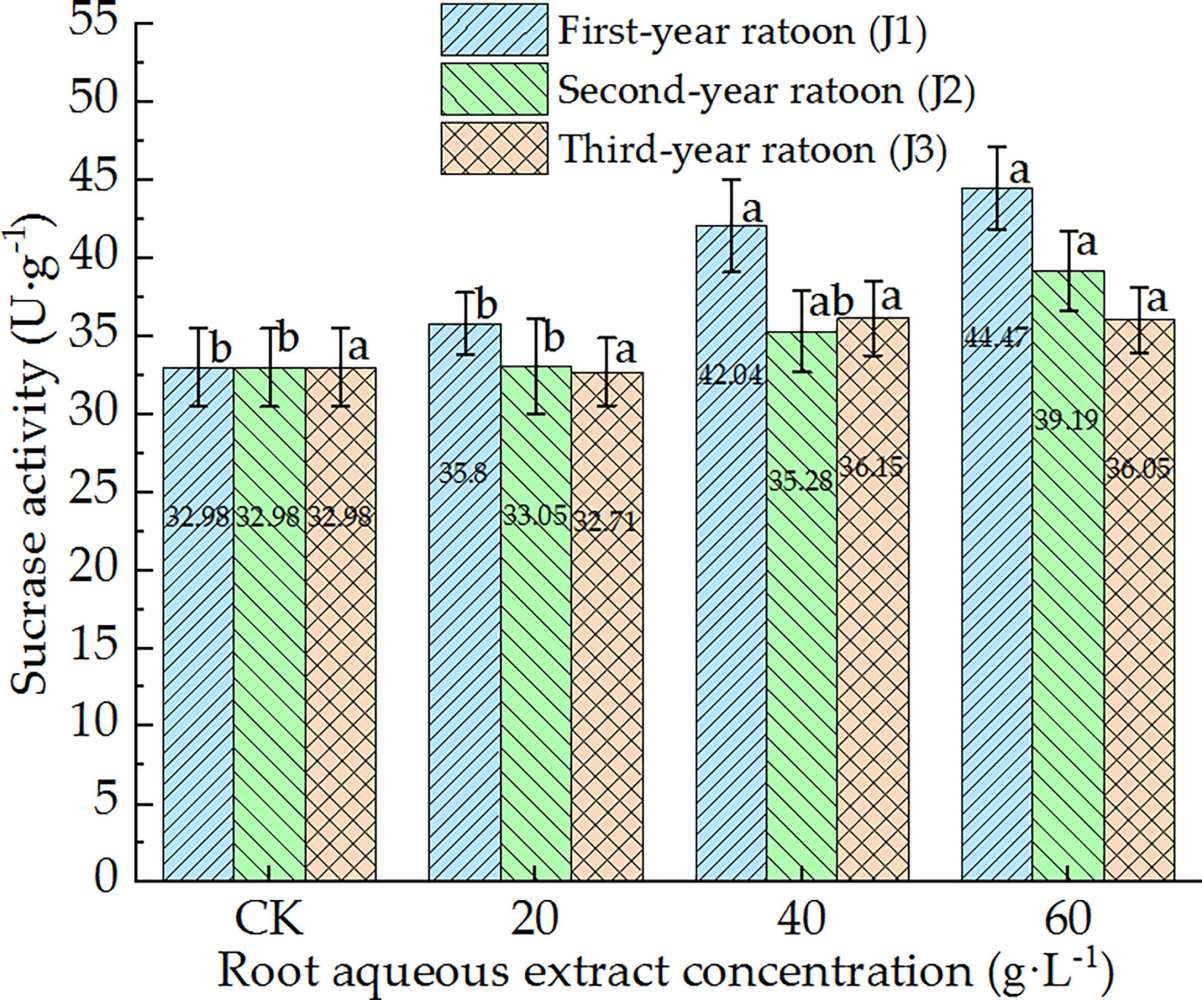
Response of SC activity in the culture medium of sugarcane seedlings to different root aqueous extracts. Different lowercase letters represent significant differences at the 5% level between different concentrations of root aqueous extract with the same ratoon age.

As shown in **Figure 9**, SC activity in the culture medium increased with increasing extract concentration and showed a general downward trend with increasing ratoon age. SC activity was significantly greater than that in CK, J1-1, and J2-1 (*P <* 0.05) only under J1-2, J1-3, and J2-3, and was lower than that in CK (non-significant difference) under J3-1. No significant differences were observed among the different concentrations when treated with third-year extracts. J1-3 led to the highest SC activity (134.8% of that in CK). Therefore, SC activity in the culture medium generally exhibited positive responses to the root aqueous extract of sugarcane ratoons. In particular, SC activity was significantly greater than that in CK and J1-1 (*P <* 0.05) under J1-2 and J1-3.

## 4 Discussion

### 4.1 Effects of treating sugarcane seedlings with root aqueous extract on osmoregulatory substance content

Allelochemicals in the aqueous root extracts of sugarcane ratoons can control the extent of membrane lipid peroxidation in plant tissues and cells by affecting the content of osmoregulatory molecules in sugarcane seedlings. In the present study, we observed that the root exudates affected the osmotic molecules in sugarcane seedlings to different degrees. The REL of sugarcane seedlings exhibited an upward trend with increasing extract concentration; this may have been because exposure to allelopathic stress resulted in changes in the permeability of the semipermeable membrane surrounding the protoplasm, which resulted in the exudation of organic matter and salts from the cells.

The Pro content in the leaves of sugarcane seedlings increased with increasing extract concentration but that in the roots showed an initial increase followed by a decrease with increasing extract concentration. These results are consistent with those reported by Gao et al. (2009) who showed that salt stress can significantly increase the Pro content in plants. Thus, stress can promote the natural responses of plant tissues to induce the transformation and accumulation of a large amount of Pro (García de la Torre et al., 2022), thereby improving the osmoregulatory and stress alleviation abilities of plant tissues under adverse conditions and assisting in the maintenance of normal plant metabolism.

The SP content in the leaves and roots of sugarcane seedlings decreased with increasing extract concentration and showed an initial increase followed by a decrease with increasing ratoon age. SS content in the leaves and roots of sugarcane seedlings increased with increasing extract concentration but showed an initial increase followed by a decrease with increasing ratoon age. This is consistent with the results of Zhang et al. (2021) that an increase in *Cinnamomum camphora* leaf litter led to a gradual decrease in SP content and a gradual increase in SS content in three types of crops. The increase in SS content induced by treatment with the root aqueous extract of sugarcane ratoons may have been because the allelochemicals in the extract hindered the absorption of external water by the seedling roots, resulting in water stress. Thus, to alleviate the physiological abnormalities in metabolism caused by water stress, a certain amount of small-molecule organic compounds, such as Pro and SS, were produced and accumulated in the cells of seedling tissues and organs (Guo, 2021). The decrease in SP content induced by treatment with the root aqueous extract of sugarcane ratoons may have been because allelochemicals in the extract inhibited the absorption of mineral ions by the seedlings, which reduced enzyme activity and protein synthesis. Alternatively, it may have been because the entry of allelochemicals into cells led to the disruption of protein structure, causing a decrease in SP content, or allelochemicals promoted the conversion of SP into SS in seedling tissues and organs to stabilise cellular osmotic potential and enhance the capacity for stress tolerance (Wang et al., 2021a).

### 4.2 Effects of treating sugarcane seedlings with root aqueous extract on antioxidant enzyme activity and membrane lipid peroxidation product content

Importantly, MDA can be used to evaluate the extent of membrane lipid peroxidation and the antioxidant capacity of tissues and cells (Chen et al., 2022; Li et al., 2022). In the present study, the MDA content in the leaves and roots of sugarcane seedlings showed an upward trend with increasing extract concentration. This may be attributed to the greater extent of membrane lipid peroxidation in the leaves of sugarcane seedlings, which resulted in the disruption of the cell membrane and enzyme activity or to the inhibition of reactive oxygen species scavenging activity. This would mean that SOD and POD activities were insufficient to scavenge the oxygen free radicals produced in the seedlings, resulting in an increase in MDA content (Wang et al., 2021b). These findings are similar to those of a previous report on the allelopathic effects of treatment with different tissues of *Trifolium repens*, which led to an increase in the MDA content of *Dactylis glomerata* seedlings (Zhang et al., 2020).

Furthermore, SOD can scavenge oxygen free radicals, regulate the extent of membrane lipid peroxidation, and protect the cell membrane from damage in plant tissues. Our study demonstrated that SOD activity in the leaves of sugarcane seedlings showed a trend of initially increasing followed by a decrease with increasing extract concentration, whereas that of the roots showed an increasing trend. Furthermore, SOD activity in both the leaves and roots showed a downward trend with increasing ratoon age. A study concerning the effects of *Trifolium repens* stem and leaf extracts on Chinese cabbage and *Viola yedoensis* Makino seedlings found that SOD activity showed a decreasing trend with increasing extract concentration (Zhang and Zhang, 2011), which differs from our results that showed SOD activity being promoted at low extract concentrations but inhibited at high concentrations. The decrease in SOD activity in the tissues of sugarcane seedlings may gradually enhance the inhibition of their growth, which may be one of the mechanisms by which seedling antioxidant enzymes adapt to allelopathic stress (Prommaban et al., 2022).

Our findings revealed that the POD activity in the leaves of sugarcane seedlings showed a downward trend with increasing extract concentration, whereas that of the roots showed an upward trend. In addition, CAT activity in the leaves of sugarcane seedlings decreased with increasing extract concentration. As for the roots, CAT activity showed a trend of gradually increasing. This may be related to the effects of allelochemicals in the extract on the activities of different antioxidant enzymes in seedling tissues or the extent, speed, and sequence of responses by different seedling tissues to the allelochemicals in the extract. For example, the extract may directly activate the root vigour of sugarcane seedlings, enhance the activities of protective enzymes in the roots, and reduce the accumulation of harmful substances in the roots, thereby protecting its cell membrane function (Yang, 2016). These findings also suggest that the various physiological indicators in plant tissues have formed different adaptive mechanisms in response to environmental stress (including allelopathy) over the course of plant evolution (Ghalati et al., 2020).

### 4.3 Effects of treatment with root aqueous extract on the photosynthetic physiological characteristics of sugarcane seedlings

Plant allelochemicals can exert a variety of physiological effects that can adversely affect the photosynthetic, physiological stress resistance, and other characteristics of plants. We found that treating sugarcane seedlings with different concentrations of root aqueous extracts led to varying degrees of inhibitory effect on the total chlorophyll content and *T*_r_ of the leaves, such that the inhibitory effect increased with increasing extract concentration. This may have been due to the decrease in the active components of key photosynthetic enzymes (e.g., SP), which disrupted the synthesis of various chloroplast pigments in seedling leaves (Zhou et al., 2009). Alternatively, this may have been due to the destruction of chloroplast lamellar structure by allelochemicals in seedling leaves, which had an inhibitory effect on the expression of photosynthesis genes in plant cells, thereby achieving the targeted suppression of chlorophyll formation (Li et al., 2021). In addition, *T*_r_ was significantly lower than that in CK (*P <* 0.05) for all ratoon ages when treated with 60 g·L^−1^ root aqueous extracts. This decrease in *T*_r_ may have been due to partial stomatal closure in seedling leaves induced by allelopathic effects (Wu et al., 2012).

The intrinsic factors affecting the photosynthetic efficiency of plants can be divided into two broad categories: stomatal and non-stomatal limitations. The former mainly involve the partial closure of stomata in leaves, whereas the latter mainly exert adverse effects on the quantum efficiency of photosystem II, leading to the degradation of chlorophyll molecules and a reduction in the photosynthetic rate (Li et al., 2021a). In the present study, *P*_n_ and *C*_i_ exhibited a trend of initially increasing followed by a decrease with increasing extract concentration. In other words, they were promoted by low extract concentrations and inhibited by high extract concentrations. This may have been because allelochemicals can affect the *P*_n_ of seedling leaves by altering their stomatal factors, such as stomatal conductance and *C*_i_ (Wu et al., 2012). These findings are consistent with those of Wu et al. (2013) that decomposing *Eucalyptus grandis* leaf litter exerted significant inhibitory allelopathic effects on the chlorophyll accumulation, *P*_n_, and *T*_r_ of *Cichorium intybus* leaves. Our findings are also consistent with those of Xu et al. (2015), who found that decomposing *Juglans regia* leaf litter exerted inhibitory allelopathic effects on the stomatal opening and transpiration process of *Brassica chinensis* leaves. These responses may have been due to the different allelochemicals accumulated as exudates or released by decomposition in the roots of different ratoon ages, which can be combined with the mechanisms of photosynthetic effects by inhibiting light energy conversion, electron transfer, and photophosphorylation in the light reaction process, thereby reducing the photosynthetic rate of leaves (Zhang et al., 2016). Thus, these findings further demonstrate that the root aqueous extract of sugarcane ratoons exerted allelopathic stress on the growth of sugarcane seedlings.

### 4.4 Effects of treatment with root aqueous extract on the bio-enzymatic properties in the culture medium of sugarcane seedlings

The various enzymes found in the plant culture medium are highly sensitive to environmental stress in the rhizosphere and can rapidly respond to changes, thus serving as an early warning of changes in the rhizosphere ecosystem (Song et al., 2012; Mo et al., 2016; Liao et al., 2017; Wang et al., 2020; Li et al., 2021b). In the present study, the activities of POD, PPO, UE, and ACP in the culture medium generally exhibited a downward trend with increasing extract concentration. Our findings are similar to those reported by Zhao and Ding (2022) that the activities of antioxidant enzymes (e.g., POD and SOD) in the leaves of *Arachis duranensis* decreased significantly (*P <* 0.05) with increasing concentrations of leaf aqueous extract from *Mucuna* sempervirens. Allelopathic stress can significantly reduce the enzyme levels in the rhizosphere and culture medium, which may be related to the decrease in the level of enzyme precursors in the culture medium due to allelopathic stress, thus precluding the need for more enzymes to eliminate the toxic effects on the roots. For example, the stress-induced reduction of phosphorous-utilisation efficiency in soil may the primary reason for the lower level of phosphatase. Our findings indicated that POD, PPO, UE, and ACP activities in the culture medium generally exhibited an upward trend with increasing ratoon age, which may have been because sugarcane ratoons of different ages produced different root exudates that contained different allelochemicals. Our findings are also consistent with those of Huang et al. (2012) on the effects of decomposing *Cinnamomum septentrionale* leaf litter on the growth of *Brassica rapa chinensis*. This may have been caused by the greater release of allelochemicals due to decomposition in the first and second years, which exerted stronger allelopathic inhibitory effects. However, as allelochemicals continued to be released over the course of decomposition, their inhibitory effects gradually weakened in the third year and even became facilitatory effects. This may be attributed to the release of nutrients or significant alterations in the abundance of allelochemical types following the copious release of allelochemicals in the roots during the initial and intermediate stages.

The present study also demonstrated that SC activity in the culture medium increased with increasing extract concentration but decreased with increasing ratoon age. This may have been because the addition of decomposition products from the sugarcane roots provided a substantial source of organic matter for microbial growth in the culture medium, which promoted the metabolism of microorganisms in the culture medium and the secretion of SC by the roots to some extent. Alternatively, the release of allelochemicals by the decomposition of the sugarcane roots may have promoted the root secretion of SC (Li and Li, 2013). This is consistent with the results of Xiao (2013) who concluded that allelopathy can promote the secretion of nutrients, such as sugars, by the roots to improve the rhizosphere microbial ecology and effectively enhance soil enzyme activity. The limitations of the study were the simulation of the autotoxic effects of allelochemicals released by root system decomposition of sugarcane ratoons on the growth of sugarcane seedlings under natural conditions.

## 5 Conclusion

In this study, the allelopathic effects of root aqueous extracts from different ages of persistent roots on the cultivation of sugarcane seedlings were studied. The results showed that the allelochemicals released from the persistent roots of sugarcane may play three roles (Bortolo et al., 2018; Li et al., 2021; Zhou et al., 2021). First, allelochemicals can affect the accumulation of osmoregulatory molecules, including REL and Pro, SP, and SS. Second, allelochemicals can cause peroxidative damage to the cell membranes of sugarcane seedlings, resulting in the inhibition of chlorophyll synthesis and effect on photosynthesis, thereby hindering seedling growth. Third, by altering the activities of various enzymes in the rhizosphere, allelochemicals can act on the supply of the available nutrients (e.g., organic matter, nitrogen, and phosphorous) in the rhizosphere, ultimately affecting the growth and development of sugarcane. The results of this study increase our understanding of the mechanisms underlying allelopathic autotoxicity during the decline in the performance of sugarcane ratoons at the physiological level. In addition, the results provide baseline data for managing sugarcane ratooning. Future studies should focus on the determination of allelochemicals in sugarcane roots and their mechanism of action at the gene expression level.

## Data availability statement

The raw data supporting the conclusions of this article will be made available by the authors, without undue reservation.

## Author contributions

LL conceived the idea and designed the experiments. XW performed the experiments, analyzed the data, and wrote the paper. SW, JZ, LZ, and ZY performed the experiments. All authors contributed to the article and approved the submitted version.

## Funding

The Innovation Platform for Academicians of Hainan Province (YSPTZX202129); This research was funded by the Key Laboratory for the Green and Efficient Production Technology of Sugarcane, Guangxi Science & Technology Normal University (GXKSKYPT2021006); Laibin Talent Project for the Comprehensive Utilisation of Sugar Resources (Laibin Talent Project [2019] No. 12); Youth Scientific Research Innovation Team for the Development and Application of Green and Efficient Technology for Sugarcane Resources (GXKS2020QNTD01); Laibin Scientific Research and Technology Development Program (Lai Ke Zhuan 202413).

## Conflict of interest

The authors declare that the research was conducted in the absence of any commercial or financial relationships that could be construed as a potential conflict of interest.

## Publisher’s note

All claims expressed in this article are solely those of the authors and do not necessarily represent those of their affiliated organizations, or those of the publisher, the editors and the reviewers. Any product that may be evaluated in this article, or claim that may be made by its manufacturer, is not guaranteed or endorsed by the publisher.
